# Functional Characterization of Detergent-Decellularized Equine Tendon Extracellular Matrix for Tissue Engineering Applications

**DOI:** 10.1371/journal.pone.0064151

**Published:** 2013-05-27

**Authors:** Daniel W. Youngstrom, Jennifer G. Barrett, Rod R. Jose, David L. Kaplan

**Affiliations:** 1 Department of Biomedical and Veterinary Sciences, Virginia-Maryland Regional College of Veterinary Medicine, Virginia Tech, Leesburg, Virginia, United States of America; 2 Department of Large Animal Clinical Sciences, Marion duPont Scott Equine Medical Center, Virginia-Maryland Regional College of Veterinary Medicine, Virginia Tech, Leesburg, Virginia, United States of America; 3 Department of Biomedical Engineering, Tissue Engineering Resource Center, Tufts University, Medford, Massachusetts, United States of America; University of Minho, Portugal

## Abstract

Natural extracellular matrix provides a number of distinct advantages for engineering replacement orthopedic tissue due to its intrinsic functional properties. The goal of this study was to optimize a biologically derived scaffold for tendon tissue engineering using equine flexor digitorum superficialis tendons. We investigated changes in scaffold composition and ultrastructure in response to several mechanical, detergent and enzymatic decellularization protocols using microscopic techniques and a panel of biochemical assays to evaluate total protein, collagen, glycosaminoglycan, and deoxyribonucleic acid content. Biocompatibility was also assessed with static mesenchymal stem cell (MSC) culture. Implementation of a combination of freeze/thaw cycles, incubation in 2% sodium dodecyl sulfate (SDS), trypsinization, treatment with DNase-I, and ethanol sterilization produced a non-cytotoxic biomaterial free of appreciable residual cellular debris with no significant modification of biomechanical properties. These decellularized tendon scaffolds (DTS) are suitable for complex tissue engineering applications, as they provide a clean slate for cell culture while maintaining native three-dimensional architecture.

## Introduction

Extracellular matrix (ECM) has emerged as a fundamental tool for developing regenerative tissue prostheses [1]. Simultaneously, bioengineered scaffolds (either natural or synthetic) are critical to improving our understanding of the complex relationship between three-dimensional topographical and biomechanical environments and stem cell growth and differentiation [2],[3]. Regardless of the intended application, decellularization protocols are designed to remove cells and debris while preserving the three-dimensional organization and ultrastructure of the extracellular matrix. Biomaterials based on stem cells seeded on decellularized tissue scaffolds are emerging as exciting options for clinical therapy, by obviating the need for traditional organ transplant or autologous donation techniques which are associated with significant morbidity [4]–[7]. Moreover, the study of stem cell/matrix interactions in a native scaffold environment furthers our understanding of basic cellular behavior and stem cell differentiation.

Tendon is an important target for tissue engineering due to its frequent involvement in musculoskeletal pathology and its comparatively simple organization. Tendon tissue repairs slowly, has a poor functional endpoint after healing, and often suffers re-injury [8]. Furthermore, there is an unmet need for functional and readily integrated graft material for human patients that suffer from traumatic tendon rupture or loss [9],[10]. The horse is an excellent model for tendon research due to its pathophysiological similarities with human degenerative orthopedic disease [11]. Additionally, there is a relatively large quantity of donor tissue available compared with other model animals, as well as significant clinical demand. Equine athletes routinely function close to the mechanical threshold for tendon damage [12] in a mildly hyperthermic environment [13], resulting in cumulative cellular and extracellular breakdown as well as changes in tissue biochemistry [14]. Tendinopathy results when this deterioration exceeds the capacity for restorative remodeling [15],[16]. Since ECM components are highly conserved, potential immunogenic reactivity is minimal [17]. Furthermore, due to the high *in *vivo tensile load experienced by the equine flexor digitorum superficialis tendon (5% average strain at a speed of 7 m/s) [18], a maximal load on the order of 10 kN [12], and the low cellularity and vascularity of the tissue, it is a strong and homogeneous starting material as a source of xenogeneic scaffold material.

It has long been recognized that cells demonstrate a complex array of behaviors in response to culture on natural collagenous matrices [19]. This characteristic has been exploited in biotechnology, as natural matrices inherently possess bioactive factors that induce host-mediated healing [20],[21]. MSC differentiation pathways are dependent on the structural and chemical composition of ECM [22], as well as its mechanical properties [23]. This is particularly true for tendon progenitor cells, whose niche is dominated by extracellular proteins [24]. Therefore, successful engineering of scaffolds for orthopedic research requires the correct physical and chemical environment to induce *de novo* tissue generation. Preparation of these tissues ideally removes cellular material, but leaves behind the critical fibrillar collagen ultrastructure, as well as the majority of glycosaminoglycans (GAGs), to aid in tissue regeneration.

Successful decellularization is a tissue-dependent procedure, and optimization of protocols has until now been lacking for dense strong tendon such as equine flexor digitorum superficialis. Nevertheless, testing protocols published for other fibrous tissues could translate into an optimized protocol for this novel tissue. Low concentrations of tri(*n*-butyl)phosphate (TnBP) and SDS have shown utility in a preliminary decellularization study of rat tail tendon [25]. An analogous study of porcine diaphragm tendon revealed that TnBP caused adequate loss of cellularity and preserved ECM architecture, while other commonly implemented detergents, including Triton X-100, did not [26]. Conversely, Triton X-100, when used in combination with other methods, reportedly aided in decellularization of flexor digitorum superficialis tendons in a chicken model [27], and has also been used in combination with SDS [28],[29]. SDS has effectively removed cellular debris from connective tissues when other methods have failed [30], and has been used successfully in whole-organ [31],[32] and even multi-organ [33] decellularization. Enzymatic decellularization without detergent exposure has also been documented for certain tissues [34]–[36], and it is common practice to combine physical agitation, chemical manipulation, and enzymatic digestion to achieve a finished product [17]. It is apparent that decellularization procedures must be optimized by tissue type and donor species, and tailored to the needs of the application.

Our aim was to test several different decellularization protocols and compare their ability to decellularize equine flexor digitorum superficialis tendon while maintaining collagen ultrastructure and minimizing loss of GAG content. Our hypothesis was that a treatment protocol using SDS would remove the majority of cellular debris without reducing collagen content or compromising structural organization of the DTS, preserving scaffold topography and mechanical properties. We anticipated higher detergent concentrations would result in GAG loss, so multiple concentrations were compared against each other and against other decellularization procedures referenced in the literature. We additionally hypothesized that our DTS would be compatible with allogeneic MSC culture, resulting in no significant loss of viability or proliferative potential.

## Materials and Methods

### Experimental Design

Equine flexor digitorum superficialis tendons were aseptically harvested from the forelimbs of eight castrated male sporting horses (mean age 12.8±1.4 years) euthanized for conditions unrelated to musculoskeletal disease. All procedures were approved by the Institutional Animal Care and Use Committee of Virginia Tech. Tendons were longitudinally sectioned into 400 µm-thick ribbons using a Padgett Model B electric dermatome (Integra Lifesciences, USA). Samples were sectioned into squares approximately 3 cm^2^ in area and were randomly assigned to treatment groups. With three replicates of each control and treated scaffold per horse, a total of 144 tendon samples were required for initial characterization. Each outcome variable was performed in triplicate on samples from each of the eight horses.

Two small representative sections were removed from every scaffold sample for biochemical characterization. These sections were dehydrated overnight in an 80°C oven, weighed, and placed into low binding affinity microcentrifuge tubes (Eppendorf, USA). One portion from each sample (4.7 mg mean dry mass) was digested in 1 mL of 1 mg/mL papain [37] (Sigma, USA) for 36 hours in a heated water bath at 65°C, while the other (2.8 mg mean dry mass) was digested with 0.1 mg/mL pepsin (Sigma, USA) in 0.5 M acetic acid for 48 hours at 4°C. All concentrations were normalized to scaffold dry mass. The remaining portions of the original scaffold samples were reserved for microscopy.

Following biochemical and microscopic characterization, the optimal protocol (2% SDS) was selected for additional comparison versus the untreated tendon control, including analysis of ultrastructure, biocompatibility and cellular integration. This was performed in triplicate on material from four horses.

### Scaffold Preparation

Tendon samples were assigned to either the untreated control group and immediately frozen at −80°C, or to a treatment group consisting of one of the following detergent types: (1) phosphate buffered saline [PBS] (Lonza, USA) control; (2) 1% tri(n-butyl)phosphate [TnBP] (Aldrich, USA); (3) 1% SDS (Sigma, USA) plus 0.5% Triton X-100 (Sigma, USA) [SDS/TX100]; (4) 1% SDS; (5) 2% SDS. All detergents were buffered in 1 M Tris-HCl (Fisher Scientific, USA), pH 7.8. In a previous study, we determined that no live cells and no residual mRNA were present in tendon samples after four freeze/thaw cycles [37]. Following that procedure, samples were individually suspended in 2 mL of their assigned detergent types in six-well plates in a refrigerated shaker at 4°C for 48 hours to allow adequate perfusion of tendon matrices. Tendon samples were then rinsed in PBS six times to remove residual detergent [38] before incubation with 0.05% trypsin-EDTA (Gibco, USA) for 10 minutes and washed with water. Samples were then stored at 4°C for 24 hours with the addition of 1 µl/mL of a mammalian tissue protease inhibitor cocktail (Sigma, USA). Additional treatment steps included incubation in DNase-I (STEMCELL Technologies, Canada) for 30 minutes and 95% ethanol (Sigma, USA) for two hours at 4°C, separated with and followed by three 10-minute washes in water. Treatment steps were conducted in a gyratory shaker (New Brunswick Scientific, USA). Samples were divided for each assay as described above.

### Biochemical Characterization

DNA content, as a marker for cell debris, was quantitatively measured in the papain scaffold digests following a single ethanol-based extraction technique using a fluorometric dye, Quant-iT PicoGreen (Molecular Probes, USA) in a ratio of 170 µL working solution to 30 µL samples/standards in a 96-well plate.

Protein content in 1∶10 dilution papain digests was measured via a standard Bradford absorbance assay with Coomassie Brilliant Blue G-250 (Fisher Scientific, USA). 50 µL of each sample was combined with 200 µL reagent solution in a 96-well plate, referencing type-I rat tail collagen (Gibco, USA) as a standard.

Solubilized collagen following digestion was assessed in 100 µL aliquots of acid/salt-washed pepsin scaffold digests using a Sircol kit (Biocolor Ltd., UK), which is based on the specific binding of Sirius red to the [Gly-x-y]_n_ triple helix motif characteristic of collagen.

Sulfated GAG content in the papain digests was quantified through a spectrophotometric assay based on 1,9-dimethylmethylene blue (Sigma, USA) and compared to a standard curve of chondroitin sulfate A from bovine trachea (Sigma, USA), with a combination of 50 µL samples/standards and 200 µL DMMB solution. Data was in all cases normalized to scaffold dry weight, obtained prior to digestion.

### Microscopic Evaluation

Samples for microscopy underwent fixation in 4% paraformaldehyde and were submitted for paraffin embedding, sectioning at 5 µm, and routine histological staining (Histoserv, Inc., USA). Longitudinal cross sections were stained with hematoxylin and eosin (H&E) or Masson’s trichrome. Images were acquired using standard brightfield techniques on an Olympus IM inverted microscope.

DNA was microscopically observed in tendon samples with ethidium homodimer-1 (EthD-1) (Invitrogen, USA) [528EX, 617EM] under an AMG EVOS_FL_ digital inverted fluorescence microscope (AMG, USA). Identical brightness and exposure settings were used for each image. A similar procedure was replicated for cell culture analysis, with the addition of calcein, AM [494EX, 517EM] via an Invitrogen Live/Dead cytotoxicity assay. Cell viability over a period of four days was assessed by a combination of live/dead fluorescence and the CellTiter 96 assay (Promega, USA).

### Scanning Electron Microscopy

Samples for SEM were dehydrated in a graded ethanol series (15%, 30%, 50%, 70%, 95%, and 100%), critical-point dried in CO_2_, and sputter coated with gold. Samples were visualized in an FEI Quanta 600 FEG scanning electron microscope and representative images of scaffold ultrastructure were acquired.

### Biomechanics

Mechanical testing was conducted on duplicate samples from native tendon and DTS from four horses, with a mean gauge length of 16.15±0.29 mm, 5.79±0.14 mm width, and 439±17 µm thickness. Scaffolds underwent failure testing at a 1 mm/s extension rate parallel to the fiber orientation while submerged in a 37°C PBS bath on an Instron 3366 (Instron, USA) using a 100 N load cell and pneumatic clamps with 100-grit sandpaper. A tangent modulus of 0.003 MPa was set for 0% strain following conversion from the raw load/extension relationship. Ultimate tensile stress was measured empirically, and its corresponding strain was calculated using a fourth-order polynomial fit to the stress/strain curve. Elastic modulus was reported at the maximum first derivative of this relationship, and yield point was determined by the corresponding maximum of the second derivative.

### Biocompatibility

Tendon samples (0.4 cm^2^) from the freeze/thaw group and from the 2% SDS experimental group were dehydrated and sterilized in the graded ethanol series as above, rehydrated in sterile PBS, and tested for biocompatibility with equine MSCs according to the procedures below. For longer-term cell culture, 1×4.5 cm DTS sections were prepared in accordance with the 2% SDS-based decellularization procedure. Additionally, a section of each scaffold was soaked in 0.05% Tween-20 (Fisher Scientific, USA), and tested with a commercial limulus amoebocyte lysate-based chromogenic endotoxin assay (GenScript, USA). Values were compared to positive and negative controls, and referenced to a broad-range standard curve of endotoxin.

### Cell Culture

Bone marrow aspirate was obtained from the sternum of a 3 year old male horse and processed using routine stem cell separation procedures [37] that were approved by the Institutional Animal Care and Use Committee. Bone marrow-derived MSCs were cultured in low-glucose GlutaMAX DMEM with 110 µg/mL sodium pyruvate (Gibco, USA) supplemented with 10% MSC FBS (Gibco, USA) and 100 U/mL sodium penicillin, 100 µg/mL streptomycin sulfate (Sigma, USA) at 37°C, 5% CO_2_, and 90% humidity. Cells were expanded and passaged twice at 80% confluence, then 250,000-cell aliquots were directly seeded over the longitudinal surface of the scaffolds in a 200 µL meniscus of the same media. Samples were then incubated for 48 hours, after which scaffolds were transferred to fresh containers. Adhesion efficiency was assessed by performing a manual count of non-adherent cells in a hemocytometer following scaffold transfer and trypsinization of the culture wells. A CellTiter 96 assay was performed for to quantify viable cells after four days. A live/dead cell staining kit was also used to visualize scaffolds using fluorescence microscopy on day four as described above.

Cellular integration within engineered tissue constructs was examined after an 11-day culture period. An autologous MSC/DTS pair from a 9 year old female horse was cultured at low density (20,000 cells/cm^2^) in the same media as previously described with the addition of 35.7 µg/mL ascorbic acid and clamped on both ends. The sample was incubated for a seeding period of 3 days followed by 8 days of immersion in media, with the solution changed every 3 days. At the experimental end point, the sample was collected and analyzed histologically to assess cellularity deep in the scaffold.

### Statistical Analysis

Quantitative data is presented as mean ± standard error for all reported assays. Differences across treatment means for each of the eight research horses were assessed using one-way repeated measures multivariate analysis of variance (MANOVA). Data was grouped by significance with P≤0.05 via alphabetical notation in applicable figures; data points that share a letter are not statistically different from one another. Additionally, decellularized groups were individually tested against the untreated controls for each of the biochemical, mechanical, and cytocompatibility outcome variables with a one-way *t*-test. Statistical significance was declared for those values that were found to meet the criteria of α≤0.01 and β≤0.02, noted with an asterisk. All analyses were performed using the commercial software programs JMP 9 (SAS Institute Inc., USA), Excel 12 (Microsoft, USA), and Mathematica 8 (Wolfram, USA).

## Results

### 2% SDS Removes Cellular Debris from Tendon Explants

**Figure 1:**
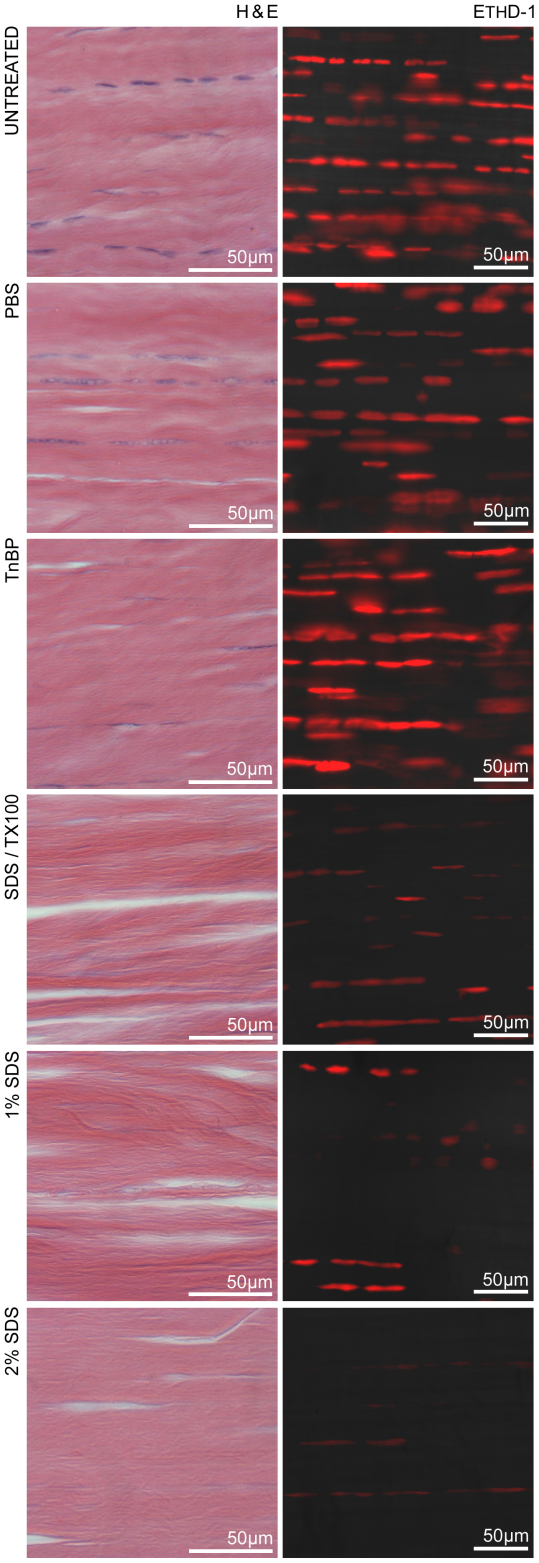
Loss of nuclear content following decellularization. Brightfield images of H&E-stained histological cross-sections (5 µm-thick) and fluorescence micrographs of scaffolds imaged with EthD-1 (400 µm-thick). Marked loss of DNA content is evident in SDS-decellularized experimental groups, with 2% SDS resulting in the most dramatic decellularization. All images are representative and were acquired from the same horse for ease of comparison.

Histological sections stained with H&E are displayed alongside equivalent EthD-1 labeled scaffolds in the first figure. Untreated tendon histology shows tenocyte nuclei exhibiting a characteristic elongated morphology, in parallel alignment with collagen fibrils in lacunae. Nuclear remains of resident tenocytes are evident, as blue staining in the H&E sections, and as red fluorescence following EthD-1 hybridization due to membrane disruption from repetitive freeze/thaw cycles. A marked reduction in DNA content was demonstrated in all three SDS experimental groups (Figure 1). Quantitative analysis of these data was performed using Image J software (National Institutes of Health, USA), and this difference was statistically significant (data not shown). Most notably, the 2% SDS decellularized group retained only 0.11±0.05 µg/mg residual DNA compared with 0.67±0.12 µg/mg in untreated tendon. Subjectively, no major alterations in scaffold architecture were noted in histological samples.

### Tendon Composition is not Significantly Altered by Decellularization

**Figure 2:**
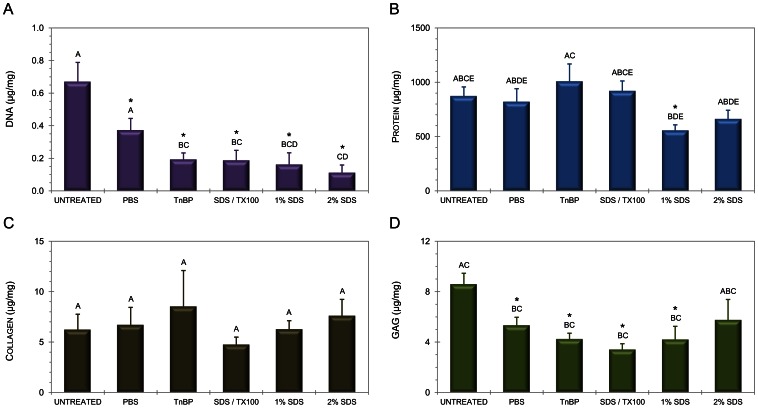
Treatment alters tendon matrix composition. Biochemical analysis of tendon scaffolds, including DNA (A), total protein (B), soluble collagen (C), and GAG content (D). Statistical significance between untreated control and treatment is annotated by use of an asterisk. Statistical differences between treatments are indicated by different letters (repeated measures MANOVA).

Tendon samples were assessed for the following biochemical outcome variables: DNA, protein, soluble collagen, and GAG levels (Figure 2). All detergent-treated groups exhibited a statistically significant reduction in DNA versus untreated tendon. The 2% SDS treatment reduced DNA content to 16.8±6.9% of untreated tendon, and this reduction was significant (p = 0.0081 with 0.0023 sphericity, power of >0.999). There was some protein loss in the SDS-treated samples, reaching significance (p<0.001) in the 1% concentration group and approaching significance (p = 0.0052) in the 2% group. There was no significant difference between SDS treatment groups (p = 0.30). The majority of GAG content was preserved. The 2% SDS decellularized tendon resulted in a small reduction in GAG content (2.83±1.64 µg/mg decrease) with p = 0.042 and a power of 0.86.

### Decellularized Tendon Maintains Desired Ultrastructure

**Figure 3:**
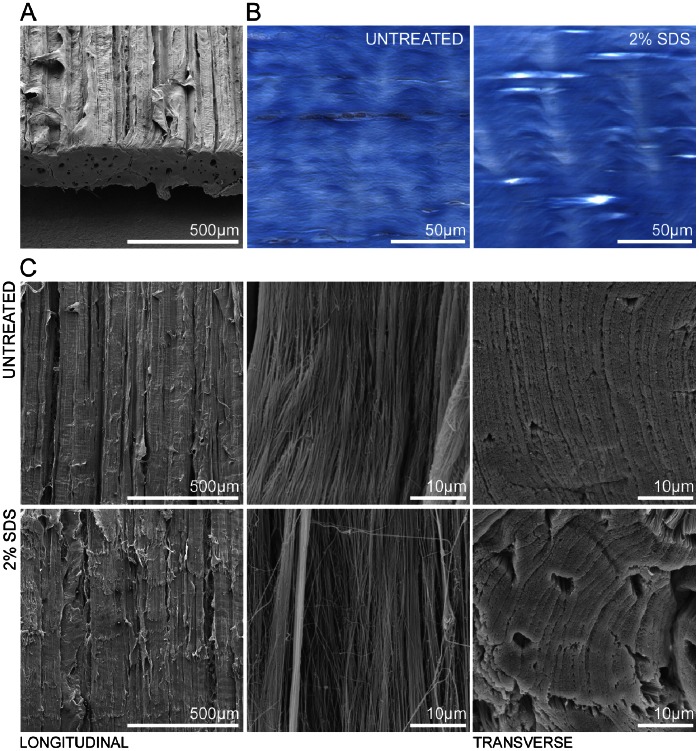
Scaffold ultrastructure. (A) Scanning electron micrograph of untreated tendon strip, angled to show both longitudinal and transverse section architecture. (B) Histological sections stained with Masson’s trichrome indicate maintenance of collagen content with a slight increase in porosity. (C) Comparison of SEM images obtained from untreated and 2% SDS decellularized tendon samples, shown at 100x and 5000x magnifications longitudinally, and 5000x transversely. Collagen ultrastructure is not adversely altered by detergent treatment.

Because the 2% SDS treatment maintained collagen content and resulted in minimal GAG loss and maximal DNA removal, this treatment was chosen for further characterization. Untreated tendon (Figure 3A) underwent structural comparison with scaffolds decellularized with 2% SDS. The characteristic deep blue coloration of collagen after staining with Masson’s trichrome was apparent even in decellularized tendon, consistent with maintenance of collagen content (Figure 3B). Samples were also evaluated via scanning electron microscopy (Figure 3C), revealing no microscale topographical differences in tendon structure aside from a subjective increase in porosity as observed in the transverse plane. No statistically significant alterations in tensile properties were observed between native equine tendon and 2% SDS decellularized scaffolds. A summary of biomechanical outcome variables is included in Table 1.

**Table 1:** DTS biomechanics.

	Native	DTS
Yield Strain (%)	8.337±0.142	7.829±0.442
Failure Strain (%)	8.712±0.510	8.851±0.216
Yield Stress (MPa)	5.774±0.440	5.782±0.775
Failure Stress (MPa)	6.029±0.414	6.045±0.759
Elastic Modulus (MPa)	76.13±4.12	70.31±5.91

Tensile testing indicated no statistically significant alterations in scaffold mechanics following decellularization. Data represents mean ± standard error values.

### Scaffolds are Biocompatible and Free of Endotoxin

**Figure 4:**
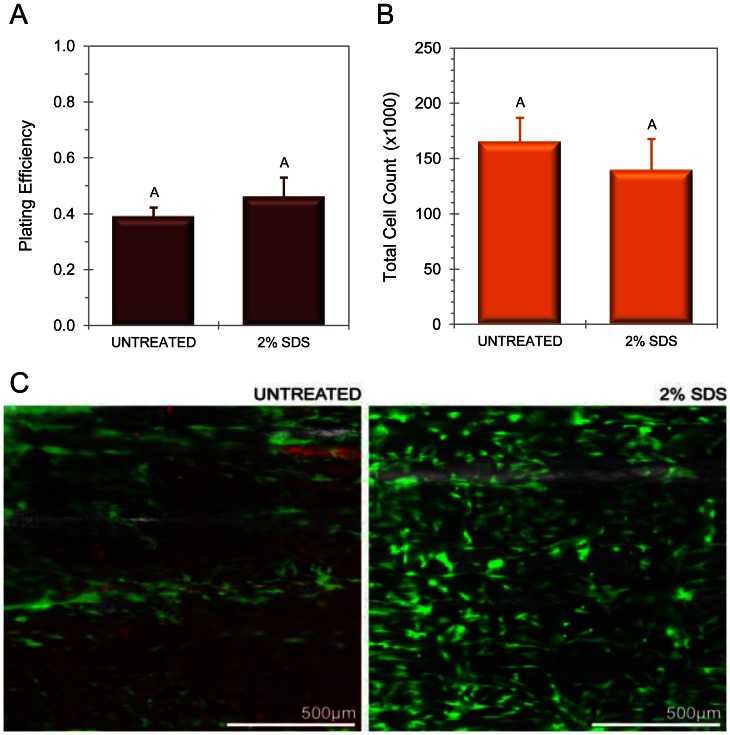
Biocompatibility as assessed with MSC culture. (A) Plating efficiency calculated as a ratio of MSCs adherent to scaffolds following a 48-hour incubation period. (B) Total cell count at 96 hours obtained via MTS assay, indicating no significant reduction in proliferation or metabolic activity on DTS. (C) Fluorescence micrographs portraying representative samples of untreated and decellularized tendon, with live cells stained green (calcein) and dead cells stained red (EthD-1).

Scaffolds decellularized with 2% SDS were compared to untreated tendon for assessment of biocompatibility (Figure 4). Prior to testing, all samples were screened and tested negative for endotoxin (data not shown). There was no significant difference in seeding efficiency between decellularized tendon and untreated tendon scaffolds; 45.9±7.0% of plated cells adhered to decellularized scaffolds compared with 38.9±3.3% for untreated tendon (p = 0.77). Cell counts on day four post-seeding indicated a global mean cell density of 420,000±17,600 cells per square centimeter of scaffold with no statistical significance between untreated and 2% SDS treated tendon. Fluorescence micrographs on day four using a live/dead cell staining kit demonstrated a healthy population of MSCs with no detectable cell death (Figure 4C). The untreated tendon had background staining of DNA, as expected. Subjectively, seeded cells exhibited an elongated morphology in parallel with the aligned scaffolds.

### MSCs Proliferate on and Penetrate Deep into DTS

**Figure 5:**
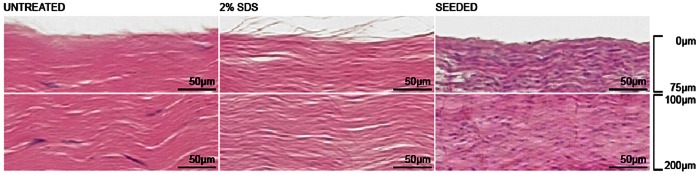
Cellular integration. H&E staining demonstrates infiltration of MSCs deep into DTS following 11 days of static tissue culture.

Culturing autologous MSCs on DTS over 11 days demonstrated marked cellular proliferation and integration, as visualized histologically (Figure 5). This strongly supports the suitability for DTS as a platform for tissue engineering applications.

## Discussion

DTS provides a structurally similar physical environment to native tendon with minimal residual cellular debris. Engineered scaffolds including DTS are geared to provide a tunable microenvironment in which we may study fundamental development as well as cell-mediated mechanisms of tissue regeneration. Additionally, advanced scaffolds such as DTS may prove valuable in extending the phenotypic stability of tenocytes [39] and tendon stem cells [40], which experience drift over extended culture periods [41]. Our novel protocol resulted in formation of biocompatible, acellular constructs that will prove valuable in this pursuit. There are several ECM-based scaffolds on the market for clinical use, but most have demonstrated limited clinical efficacy as standalone products [7] and no tissue-specific commercial options for tendon and ligament engineering or augmentation are available from animal sources [42]. Moving forward, optimization of tendon ECM decellularization focusing on structural stability and accessibility to cell seeding are crucial to the development of useful reconstructive scaffold materials [43]. Equine DTS represents an improvement to current competing technologies in that regard, as it provides a unique combination of long graft length, mechanical strength, and inexpensive small-scale production.

We evaluated the effect of several candidate detergents in combination with physical and enzymatic cell disruption, followed by a series of wash steps and sterilization in ethanol, on longitudinal tendon ribbons. These sections were acquired from the core regions of tendon explants to take maximum advantage of intrinsic properties including comparatively low cell density and vascularity as well as uniform ultrastructure and mechanics compared with other regions such as epitenon [44]–[46]. As 300 µm is reported as the minimum desirable tendon scaffold thickness from a biomechanical standpoint [47], we selected a thickness of 400 µm to compensate for potential loss of surface ultrastructure following decellularization. This thickness allows complex tissue construction techniques such as stacking [48], rolling [49], and forming composites [50], yet is strong enough to facilitate early range of motion. And, it would allow for repair of partial tears, and replacement of small tendons such as flexor tendons of the hand via a rolling or stacking technique. Scaffolds underwent rigorous comparison with validated methods for efficacy of removal of cellular debris, maintenance of ECM composition and functional properties, and compatibility for allogeneic MSC culture. 2% SDS treatment, in combination with trypsinization, treatment with DNase-I, and ethanol sterilization, resulted in optimal characteristics.

DNA was selected as a sensitive indicator of cell debris due to its high stability, and common use as a proxy for other cellular debris. While DNA is not cytotoxic, it is a useful index that strongly correlates with adverse host immunogenicity [51]. EthD-1 was selected as a marker for nuclear content following confirmation that no live cells or mRNA remained after four freeze/thaw cycles [37]. The data indicates that 2% SDS removed a mean 83% of DNA from the tendon scaffolds, and 1% SDS resulted in a 76% reduction. This represents an improvement over other effective published decellularization protocols for tendon (67% [27]). SDS in combination with Triton X-100 removed 72% of DNA, and TnBP was the least effective detergent-based method with 71%. Detergent-free decellularization (PBS group) removed only 44% of nuclear content. These numbers correlate with fluorescence images of intact scaffolds. However, it is interesting to note that residual TnBP may enhance EthD-1 fluorescence, as the intensity displayed on the representative micrograph does not correspond to results obtained with complimentary techniques, including H&E staining.

The Bradford assay demonstrated a mean 24% reduction of total protein, which may correspond to loss of soluble intracellular proteins, chromosomal proteins such as histones, organelle proteins such as lysosomal proteases and/or noncollagenous extracellular matrix proteins. The statistical significance of the protein loss following 1% SDS decellularization most likely corresponds to intracellular proteins or non-collagenous matrix proteins, as soluble collagen content following DTS digestion was statistically unchanged with all experimental groups failing to approach any of the statistical significance criteria.

It is interesting to note that our procedure leaves biologically relevant residual GAG content, as experiments in porcine ACL demonstrated that SDS decellularization dramatically reduced sulfated GAG content compared with untreated tissue [52]. Maintenance of GAG content is desirable due to the role of the molecules in signal modulation, water composition, and tissue biomechanics [53]. The DMMB assay demonstrated a 33% reduction in GAG content which approached statistical significance (as it reached in other experimental groups), but residual GAG content was much higher than anticipated. Statistical differences between the 1% and 2% SDS-treated groups in protein and GAG outcomes may be attributed to the relatively small sample size. A canine tendon decellularization protocol that did not use detergent has been shown to maintain native proteoglycan content [36], but our data indicates that equine tendon requires detergent decellularization, which may fortunately still be accomplished while preserving the integrity of functional macromolecules. This highlights the necessity for tissue-specific optimization of decellularization protocols.

Scanning electron microscopy allowed analysis of potential ultrastructural modifications to decellularized scaffolds. Subjectively, SEM images did not demonstrate disruption of collagen alignment or fiber thickness at the micrometer level, though a slight reduction in fibril density was evident in the SDS-treated group that may prove beneficial for seeding scaffolds with cells. This may correspond with the 18% increase in seeding efficacy noted in subsequent cell culture, as the increase in porosity and surface area was hospitable toward MSCs. Histological sections stained with Masson’s trichrome revealed similar staining of ECM, indicative of native collagen composition, while also suggesting a subtle increase in porosity, supporting the electron micrographs.

Biomechanical parameters of DTS did not significantly deviate from control tissue, contrary to results reported in other decellularization protocols [54]. An insignificant extension of the toe region and a decrease in yield strain was observed (p = 0.16) with a slight increase in elastic modulus (p = 0.11), indicating trends that may emerge with a larger sample size. Interestingly, these data suggest the potential for safe elongation protocols up to 7% strain before plastic deformation is observed, with an ideal linear extension region centered on 4.1±0.2% strain (data not shown). This characterization is necessary for experiments involving mechanical manipulation of cell-seeded constructs.

While endotoxin contamination has not been associated with long-term derogative effects in tissue graft integration or remodeling [55], testing is essential for evaluation of biomaterials destined for bioreactor cell culture and medical device applications [56]. Our DTS contained no detectable levels of endotoxin. In order to assess the initial feasibility of using DTS as a raw material for tissue engineering, we seeded our scaffolds with MSCs for four days due to their frequent use in cell-based orthopedic tissue engineering studies, and the possibility that autologous MSCs could be used on DTS as a therapeutic graft material [57]. MSC culture was successful in terms of plating efficiency, cell proliferation, and viability. As stated, DTS had a higher seeding efficiency than identically sectioned control tissue, perhaps due to increased porosity resulting from the decellularization process. For viability, no dead cells were seen adherent to the scaffolds among 2% SDS DTS samples. A statistical comparison of cell viability on DTS versus control tissue could not be performed due to the presence of high background staining of endogenous dead cells in non-decellularized control tendon. The influence of long-term culture on the growth and differentiation of MSC-seeded DTS constructs has only begun to be explored, and will be the subject of further investigation. However, the results of an 11-day study of autologous MSC/DTS constructs showed excellent cellular integration throughout the depth of the scaffold and further supports its lack of cytotoxicity as a scaffold material.

Acellular ECM represents a versatile, biocompatible scaffold for three-dimensional cell culture. This study validated a novel natural scaffold for tendon tissue engineering purposes. Equine flexor digitorum superficialis tendon is well suited for use in tissue engineering applications, bioreactor cell culture studies and graft material as a result of its robust mechanical properties, homogeneous low cellularity, low vascularity, and long length and width. Our protocol, based on 2% SDS detergent decellularization in conjunction with free/thaw lysis, trypsinization, DNase-I digestion, and ethanol sterilization induces practical acellularity without compromising functionality. The remaining extracellular matrix material maintains the biochemical composition, ultrastructure, and mechanics of native tendon, yet has minimal residual cellular debris. This provides a clean slate for subsequent cell culture, allowing exploitation of the features intrinsic to physiological tendon matrix without concern over functional or immunological interference from the original resident cells.
